# Insights from the first IOC Olympian Health Cohort: injury and illness in Olympians preparing for the Tokyo 2020 Summer and Beijing 2022 Winter Olympic Games

**DOI:** 10.1136/bmjsem-2025-002545

**Published:** 2025-09-21

**Authors:** Debbie Palmer, Torbjorn Soligard, Gwen Fernandes, Dave Collins, Niall Elliott, Paul Kelly, Iain R Murray, Lars Engbretsen

**Affiliations:** 1 Edinburgh Sports Medicine Research Centre, University of Edinburgh Institute for Sport Physical Education and Health Sciences, Edinburgh, UK; 2 UK Collaborating Centre on Injury and Illness Prevention in Sport, University of Edinburgh, Edinburgh, UK; 3 Medical and Scientific Department, International Olympic Committee, Lausanne, Switzerland; 4 Academic Rheumatology, School of Medicine, University of Nottingham, Nottingham, UK; 5 Sports Medicine, Sport Scotland Institute of Sport, Stirling, UK; 6 Physical Activity for Health Research Centre, University of Edinburgh Institute for Sport Physical Education and Health Sciences, Edinburgh, UK; 7 Department of Trauma and Orthopaedics, The University of Edinburgh, Edinburgh, UK; 8 Department of Orthopaedic Surgery, Oslo University Hospital, Oslo, Norway

**Keywords:** Injury, Illness, Olympics, Health promotion

## Abstract

**Objective:**

To describe the prevalence and nature of Olympic-career related injuries and illnesses, and behaviours during injury/illness, in Olympians in the 4 years prior to their participation at the Tokyo 2020 and Beijing 2022 Olympic Games.

**Methods:**

315 current Olympians from 70 countries completed a cross-sectional online survey, distributed by direct email through National Olympian Associations and World Olympians Association databases. Questions included Olympic sport exposure, significant training and competition injury and illness history (lasting >2 weeks) and athlete behaviours during injury/illness.

**Results:**

65% of Olympians were women (35% men), representing 51 sports (37 summer, 14 winter), aged 28.6 years (4.6). Overall, 58.5% (95% CI 52.2% to 64.9%) of summer and 55.6% (95% CI 44.7% to 66.4%) of winter Olympians were injured, with knee injuries most frequent (19.6% summer, 27.8% winter Olympians). Injury rates were similar between males and females. 17.1% (95% CI 12.3% to 21.9%) of summer and 23.5% (95% CI 13.1% to 31.3%) of winter Olympians were ill, with respiratory illness most frequent. Illness rates were (non-significantly) higher for female versus male winter Olympians (adjusted relative risk 2.04 (95% CI 0.73 to 5.76)) but similar between male and female summer Olympians. 78% of Olympians said they put the most pressure on themselves to return from injury/illness quickly. Almost half reported using painkillers during injury, while one-quarter continued full training/competition during injury/illness. Injury and illness prevalence followed similar bimodal and trimodal seasonal patterns for summer and winter Olympians, respectively.

**Conclusions:**

Olympians report a significant history of injury and illness across the 4 years before the Olympic Games. A biopsychosocial approach that supports athletes during injury/illness absences is needed. Concurrent injury/illness prevention strategies should be considered to reduce the burden of both injuries and illnesses at key times in an athlete’s season.

**WHAT IS ALREADY KNOWN ON THIS TOPIC**

- Sport and exercise may confer health benefits, but elite sport is also associated with an increased risk of injury, illness and other health issues.
- There is detailed information on the occurrence of injuries and illnesses in Olympians during the Olympic Games, and a growing understanding of retired elite athlete health.
- However, methodological limitations to these studies mean there are gaps in our understanding of Olympian health—both in the periods between Olympic Games and in later life.

**WHAT THIS STUDY ADDS**

- This is the first longitudinal, global study of Olympian self-reported injuries and illnesses during the 4-year cycle up to a Summer and Winter Olympic Games.
- Olympians present with a significant burden of injury and illness, with nearly two-thirds of Olympians reporting a significant (>2 weeks) injury and one in five a significant illness in the preceding 4 years.
- Olympians put the greatest pressure on themselves to return to sport as quickly as possible, with nearly half taking painkillers, and a quarter continuing all training and competition, during injury/illness.
- Similar, seasonal injury/illness patterns for Summer and Winter Olympians suggest that the occurrence of injuries and illnesses may not be independent of one another.

**HOW THIS STUDY MIGHT AFFECT RESEARCH, PRACTICE OR POLICY**

- When supporting athletes during injury or illness-related absences, it is important to address athlete behaviours that may impact on their recovery and to consider a biopsychosocial approach that includes all of the multidisciplinary support team.
- The results of this study suggest that concurrent injury/illness prevention strategies could help to reduce the burden of both injuries and illnesses at key times in an athlete’s season.

## Introduction

 Sports injury and illness prevention, and the protection of athlete health, are key mandates for the International Olympic Committee (IOC).^1^ Sport and exercise may confer many health benefits; however, sport participation also carries an associated increased injury and illness risk, particularly at the elite level.^2^,^5^ Much is known about the occurrence and nature of elite athlete injuries and illnesses during major sporting events, for example during the Olympic Games and World Cup/Championship events within football (soccer), rugby, athletics and swimming.^2^,^5^ Recent IOC in-Games surveillance studies have reported 9%–10% of athletes were injured and 4% were ill at the Tokyo 2020 Summer and Beijing 2022 Winter Games.^6 7^ While in-game surveillance studies continue to provide important ongoing information, they are limited to data capture during a 3-week window, once every 4 years.^6 7^

In a recent retired Olympian study, 63% of Olympians reported having had a significant injury at some point during their sport career,^8^ and these risks can exert a longer term, even lifelong, impact. Significant joint injury is a risk factor for the development of osteoarthritis (OA) in the general population, and there is evidence from retired athletes in football, rugby and Olympic sports pointing to an association between joint injury, ongoing pain and the development and progression of OA.^9^,^12^ However, despite higher rates of pain and post-traumatic OA, retired athletes report better general health in later years when compared with the general population.^9^,^13^ These retired athlete studies provide important new knowledge; however, most studies are cross-sectional and there are limitations in terms of recall bias due to the retrospective nature of athlete health questions. Additionally, they offer limited information on temporality or causality, so our understanding of the risk factors associated with some health issues athletes face may also be limited.

Currently, there are significant gaps in our understanding of Olympian health, and what happens between those health issues reported during the Olympic Games and information gathered when athletes get to retirement.^6^,^8^ The IOC Olympian Health Cohort was designed to recruit and establish a cohort of current Olympians competing in the Summer and Winter Olympic Games, to prospectively follow them on a range of health and well-being issues, throughout their Olympic careers and into retirement.^14 15^ Prospective monitoring will counter some of the methodological issues associated with previous studies and allow a more timely and accurate understanding of what happens to them across their careers.

In this paper, we present the first data from the new IOC Olympian Health cohort.^15^ We aimed to describe the (1) characteristics of the newly recruited IOC Olympian Health Cohort, (2) prevalence and nature of their Olympic career-related injuries and illnesses and (3) treatments and behaviours during injury and illness, in the 4 years prior to the Tokyo 2020/2021 and Beijing 2022 Olympic Games.

## Methods

This study was a cross-sectional study collecting self-report survey data on Olympians. The study protocol, including methods for recruitment of the cohort and questionnaire development, is previously described.^15^ The survey was available in eight languages (English, French, Spanish, German, Russian, Chinese, Korean and Japanese). Olympians who competed at the 2020/2021 Tokyo Olympic Games and 2022 Beijing Olympic Games were eligible to participate in the study. Based on participation rates (Tokyo 11 315 athletes; Beijing 2848 athletes) across the two Olympic Games, this equated to 14 163 (6699 female; 7464 male) eligible athletes.

### Recruitment

Promotional video materials were developed, featuring current well-known Olympians as study advocates, and these were promoted on social media prior to the study launch. Recruitment occurred between August 2022 and July 2023, where a password-protected survey link was embedded in study invitation emails sent directly to Olympians. Emails were distributed through IOC platforms athlete365, Olympic Athlete Hub, Athlete Learning Gateway databases. Recruitment also occurred via National Olympic Committees (NOCs) and National Olympians Associations. Finally, Olympians were able to directly sign up for the study via the dedicated study website, where their eligibility was confirmed.

### Questionnaire

Using an online web-based survey (hosted by Qualtrics) the questionnaire was developed by the IOC Cohort research group using existing validated questionnaire tools and following the IOC consensus statement on methods for recording and reporting injuries and illnesses in sport.^16^ The questionnaire asked questions on (1) demographics (eg, age, stature, education); (2) Olympic sport exposure (years, level of participation, hours training and competing); (3a) Significant injury history for example, Olympic sport-related and ‘other’ injuries (other sport or non-sport) over the preceding 4 years (up to their Olympic Games participation) and (3b) significant illness history, over the preceding 4 years—including treatments and behaviours during injury/illness.^15^

### Definitions

For the purposes of this study, Olympians included those who had competed in Tokyo 2020/2021 or Beijing 2022.^15^ A significant Olympic career sport-related injury/illness was defined as ‘any injury/illness that occurred during training or competition that impacted the athlete’s ability to continue to train and/or compete for most days for at least 2 weeks’. Injuries or illnesses lasting less than 2 weeks were not recorded. Injury/illness severity was defined by the estimated total number of days between the injury/illness and the athletes’ full return to sport participation.

### Patient and public involvement

A patient advisory group of current and retired Olympians provided input into the questionnaire content and design including questionnaire length and acceptability. Comments were incorporated into the final version of the questionnaire. At the end of the study survey, Olympians were provided an opportunity to give feedback on questionnaire topics via an open free text box, that is, what questions they felt were missing and would like to have been asked. The feedback provided will be used to refine future questionnaire content. Representatives of the patients (Olympians) will be invited to help inform dissemination of the study results and direction of future injury and illness prevention strategies.

### Equity, diversity and inclusion statement

Our study population included female and male athletes. The author group includes mixed genders (two female, six male), different ages and career stages (early to late career) and professional backgrounds (sports injury and illness surveillance, long-term athlete health, sport and clinical psychology, sports medicine, orthopaedics, physical activity for health, elite athlete).

### Data analysis

Analysis was conducted as outlined in the study protocol.^15^ Primary outcomes were the prevalence of injury and illness. Secondary outcomes included injury and illness treatments and behaviours. Data are presented as mean+SD for numerical variables (median and range where data are not normally distributed), and frequencies (proportion) for categorical variables. Prevalence was calculated based on the number of athletes injured/ill divided by the total number of athletes and presented as percentage (%) with 95% CIs. Differences between groups were analysed by t-test (or Mann-Whitney where appropriate) and χ² test.^17^ Relative risk (RR) (with 95% CI) between groups was calculated by Poisson regression model, assuming constant hazard per group and adjusting for a priori sport, sex and age where appropriate. Significance was accepted at p<0.05 (equal variances assumed).

## Results

### Demographics

There were 408 survey entries, and after removing blank (n=63), duplicate (17) and ineligible (13) responses there were 315 questionnaires for data analysis. This equated 204 female and 109 male Olympians (2 sex unknown), comprising 234 Tokyo 2020 Summer (from 67 countries; 37 sports) and 81 Beijing 2022 Winter (28 countries; 14 sports), Olympians (table 1, online supplemental file 1).

**Table 1:** Cohort demographics

	Tokyo 2020 Olympians	Beijing 2022 Olympians	Total
No. of athletes (male/female)	234 (77/155*)	81 (32/49)	315 (109/204)
Height (cm), mean (SD)	182.5 (8.3)/169.8 (7.7)	181.8 (5.8)/167.7 (6.8)	173.3 (9.5)
Weight (kgs), mean (SD)	81.4 (14.5)/64.0 (9.4)	82.2 (10.3)/64.4 (9.3)	71.4 (13.0)
BMI (kg/m^2^), mean (SD)	22.9 (3.1)	23.6 (2.7)	23.1 (3.0)
Age (years), mean (SD)	28.7 (4.6)	28.3 (4.8)	28.6 (4.6)
International years competing	10.2 (4.5)	10.5 (5.2)	10.3 (4.7)
No. of Olympic Games (range)	1.39 (1–5)	1.88 (1–6)	1.52 (1–6)
First time Olympians (%)	168 (72)	41 (51)	209 (66)
Olympic status—still competing/retired	187/46*	70/10*	157/56

* Sex unknown n=2; active status unknown n=2.

BMI, body mass index.

### Olympic career injury and illness

In total, there were 317 injuries in 182 Olympians, and 74 illnesses in 58 Olympians. Across the preceding 4 years up to Tokyo 2020, 58.5% (95% CI 52.2% to 64.9%) of Summer Olympians were injured and 17.1% (95% CI 12.3% to 21.9%) were ill (table 2). For Summer Olympians, injury/illness rates did not differ between females and males (injured females 60.6% (95% CI 46.9% to 74.3%) vs injured males 55.8% (95% CI 41.9% to 69.7%); adjusted RR 0.90 (95% CI 0.63 to 1.30)), (ill females 17.4% (95% CI 6.8% to 28.0%) vs ill males 16.8% (95% CI 6.4% to 27.4%); adjusted RR 1.01 (95% CI 0.52 to 1.98)).

**Table 2:** Tokyo 2020 and Beijing 2022 Olympian injuries and illnesses, by sport

	No. athletes (m/f)	Injury						Illness			
		No. of injuries (m/f)	No. of injured athletes (m/f)	Injury prevalence (%)	Injuries/ athlete/ year	Training IRR (/1000 hours)	Competition IRR (/1000 hours)	No. of illnesses (m/f)	No. of ill athletes (m/f)	Illness prevalence (%)	Illnesses/ athlete/ year
Tokyo 2020 Olympians											
Athletics	52 (15/37)	64 (13/51)	34 (8/26)	65.4	0.31	0.07	3.78	9 (2/7)	8 (2/6)	15.4	0.04
Baseball/softball	4 (2/2)	2 (1/1)	2 (1/1)	50	0.13	0.09	0.14	–	–	–	–
Beach volleyball	3 (2/1)	3 (2/1)	3 (2/1)	100	0.25	0.17	0.66	1 (1/0)	1 (1/0)	33.3	–
Boxing	3 (2/1)	–	–	–	–	–	–	1 (1/0)	1 (1/0)	33.3	0.08
Canoe slalom	4 (2/2)	3 (1/2)	2 (1/1)	50.0	0.19	–	0.59	–	–	–	–
Canoe sprint	6 (1/5)	6 (0/6)	2 (0/2)	33.3	0.25	–	4.12	1 (0/1)	1 (0/1)	16.7	0.04
Cycling*	16 (4/12)	13 (1/12)	7 (1/6)	43.8	0.20	0.14	0.41	3 (0/3)	3 (0/3)	18.8	0.05
Diving	7 (4/3)	9 (4/5)	5 (3/2)	71.4	0.32	–	6.37	1 (1/0)	1 (1/0)	14.3	0.04
Fencing	9 (2/7)	10 (1/9)	6 (1/5)	66.7	0.28	0.17	1.23	2 (0/2)	2 (0/2)	22.2	0.06
Gymnastics*†	10 (1/8)†	15 (1/13)†	7 (1/6)	70	0.38	0.04	5.54	2 (0/2)	2 (0/2)	20.0	0.05
Hockey	10 (2/8)	9 (0/9)	4 (0/4)	40	0.23	0.16	1.40	–	–	–	–
Judo	11 (4/7)	21 (8/13)	10 (4/6)	90.9	0.48	0.24	4.26	2 (1/1)	2 (1/1)	18.2	0.05
Modern pentathlon	3 (1/2)	5 (2/3)	2 (1/1)	66.7	0.42	0.11	1.92	3 (0/3)	1 (0/1)	33.3	0.25
Rowing	20 (7/13)	23 (7/16)	12 (4/8)	60	0.29	0.01	7.93	10 (4/6)	6 (3/3)	30.0	0.13
Rugby 7s	7 (5/2)	9 (8/1)	5 (4/1)	71.4	0.32	0.24	0.75	–	–	–	–
Sailing	12 (5/7)	13 (6/7)	10 (5/5)	83.3	0.27	0.07	0.90	2 (2/0)	2 (2/0)	16.7	0.04
Shooting	9 (2/7)	5 (0/5)	4 (0/4)	44.4	0.14	0.03	0.78	2 (0/2)	1 (0/1)	11.1	0.06
Sport climbing	4 (1/3)	4 (1/3)	2 (1/1)	50	0.25	0.05	1.48	–	–	–	–
Swimming†	15 (5/9)†	6 (1/4)†	4 (1/3)	26.7	0.10	–	1.37	3 (1/2)	3 (1/2)	20.0	0.05
Water polo	4 (2/2)	6 (4/2)	4 (2/2)	100	0.38	0.17	1.74	–	–	–	–
Weightlifting	4 (2/2)	5 (2/3)	3 (1/2)	75.0	0.31	–	12.63	2 (0/2)	1 (0/1)	25.0	0.13
Sport unknown	8 (1/7)	0 (0/2)	1 (0/1)	12.5	–	–	–	3 (0/3)	2 (0/2)	25.0	0.09
Total summer	234 (77/155)	245 (65/180)	137 (43/94)	58.5	0.26	0.07	1.90	52 (15/37)	40 (13/27)	17.1	0.06
Beijing 2022 Olympians											
Alpine skiing	7 (5/2)	7 (6/1)	3 (2/1)	42.9	0.25	0.17	1.02	6 (5/1)	4 (3/1)	57.1	0.25
Biathlon	6 (2/4)	5 (1/4)	4 (1/3)	66.7	0.21	0.04	2.13	3 (0/3)	3 (0/3)	50.0	0.13
Bobsleigh	6 (4/2)	10 (6/4)	5 (2/3)	83.3	0.42	0.18	4.12	2 (1/1)	2 (1/1)	33.3	0.08
Cross country skiing	15 (6/9)	10 (7/3)	8 (3/5)	53.3	0.17	0.04	0.55	6 (0/6)	4 (0/4)	26.7	0.10
Curling	10 (1/9)	3 (2/1)	2 (1/1)	20	0.08	0.07	0.10	1 (0/1)	1 (0/1)	10.0	0.03
Freestyle skiing	13 (4/9)	18 (3/15)	10 (3/7)	76.9	0.35	0.11	3.16	2 (0/2)	2 (0/2)	15.4	0.04
Ice hockey	7 (4/3)	2 (2/0)	1 (1/0)	14.3	0.07	0.05	1.08	–	–	–	–
Speed skating	5 (1/4)	8 (1/7)	4 (1/3)	80.0	0.40	–	7.58	–	–	–	–
Short track	5 (2/3)	3 (1/2)	2 (1/1)	40.0	0.15	–	1.35	–	–	–	–
Skeleton	3 (1/2)	2 (1/1)	2 (1/1)	66.7	0.17	–	6.41	–	–	–	–
Total winter	81 (32/49)	72 (33/39)	45 (18/27)	55.6	0.22	0.07	1.24	22 (7/15)	19 (5/14)	23.5	0.07
Grand total all	315 (109/204)	317 (98/219)	182 (61/121)	57.8	0.25	0.07	1.70	74 (22/52)	59 (18/41)	18.4	0.06

Sports with 2 or fewer athletes not presented (including ski jumping, nordic combined, luge, figure skating, volleyball, triathlon, karate, surfing, marathon swimming, handball, football, equestrian, basketball, badminton, archery, winter sport unknown).

* Gymnastics (artistic, rhythmic, trampoline) and cycle disciplines (bmx, mtb, track, road) are combined.

† Sex unknown for one artistic gymnastics and one swimming athlete.

IRR, injury incidence rate; m/f, male/female.

In the preceding 4 years to Beijing 2022, 55.6% (95% CI 44.7% to 66.4%) of Winter Olympians were injured and 23.5% (95% CI 13.1% to 31.3%) were ill. Injury rates between female and male Winter Olympians (female 55.1% (95% CI 41.2% to 69.0%) vs male 56.3% (95% CI 42.4% to 70.1%; adjusted RR 0.71 (95% CI 0.39 to 1.28) and illness rates (female 28.6% (95% CI 15.9% to 41.2%) vs male 15.6% (95% CI 5.45% to 25.8%); adjusted RR 2.04 (95% CI 0.73 to 5.76)), also did not significantly differ.

In analysing injuries that impacted on athletes using a>4-week significant injury definition, this equated to 49.1% of Summer Olympians and 50.6% of Winter Olympians being injured.

### Injury location, type and diagnosis

**Figure 1:**
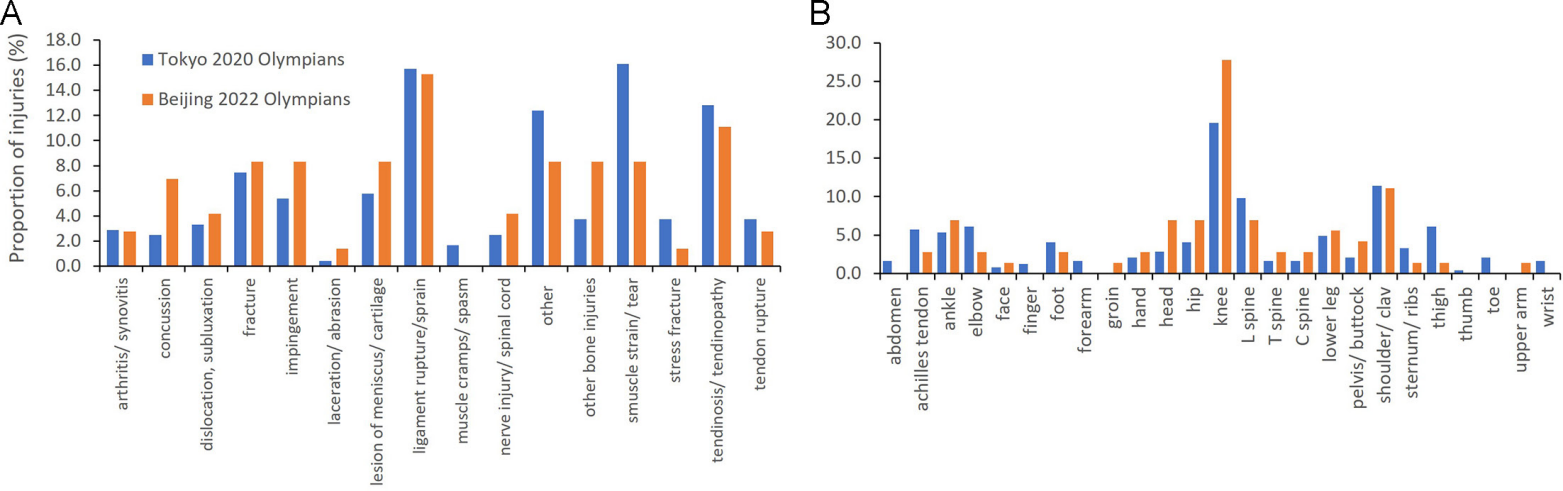
(A, B) Injury location and type for Tokyo 2020 and Beijing 2022 Olympians.

The knee was the most commonly injured body location (Tokyo Olympians 19.6% of all injuries; Beijing Olympians 27.8%), followed by the shoulder (11.4% and 11.1%) and lumbar spine (9.8% and 6.8%). Muscle strain/rupture (16.1%) and ligament injuries (15.7%) were most common for Tokyo Olympians, and ligament (15.2%) and tendinopathy injuries (11.1%) for Beijing Olympians (figure 1A,B).

Knee lesion of meniscus and ligament rupture/strains occurred predominantly in judo (n=8) and freestyle skiing (n=6). Shoulder fractures, dislocations and ligament injuries occurred in cycling (n=4) and rugby (n=3). Lumbar spine, nerve and lesion of meniscus injuries occurred most frequently in rowing (n=4).

### Illness system and cause

**Figure 2:**
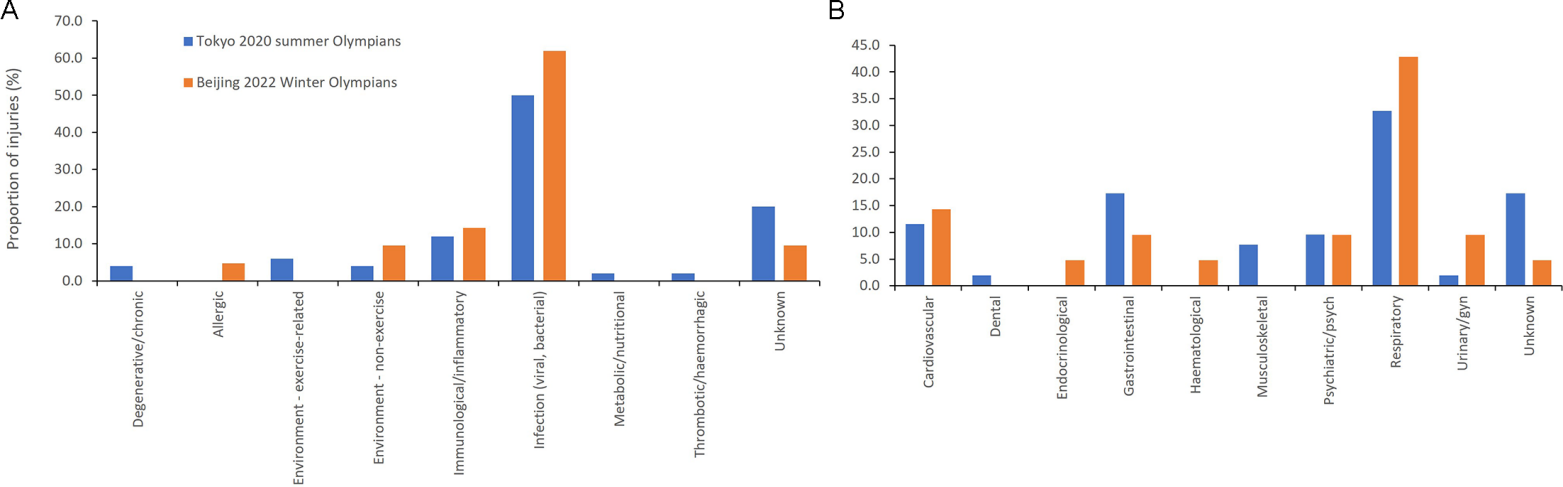
(A, B) Illness system and cause of illness for Tokyo 2020 and Beijing 2022 Olympians.

Respiratory illness was most common for both Summer (32.7% of all illnesses) and Winter Olympians (42%) followed by GI illness (17.3%) in Summer and cardiovascular illness (14.3%) in Winter, Olympians. Infection was the most common type of illness (figure 2A,B).

### Seasonal injury and illness patterns

**Figure 3:**
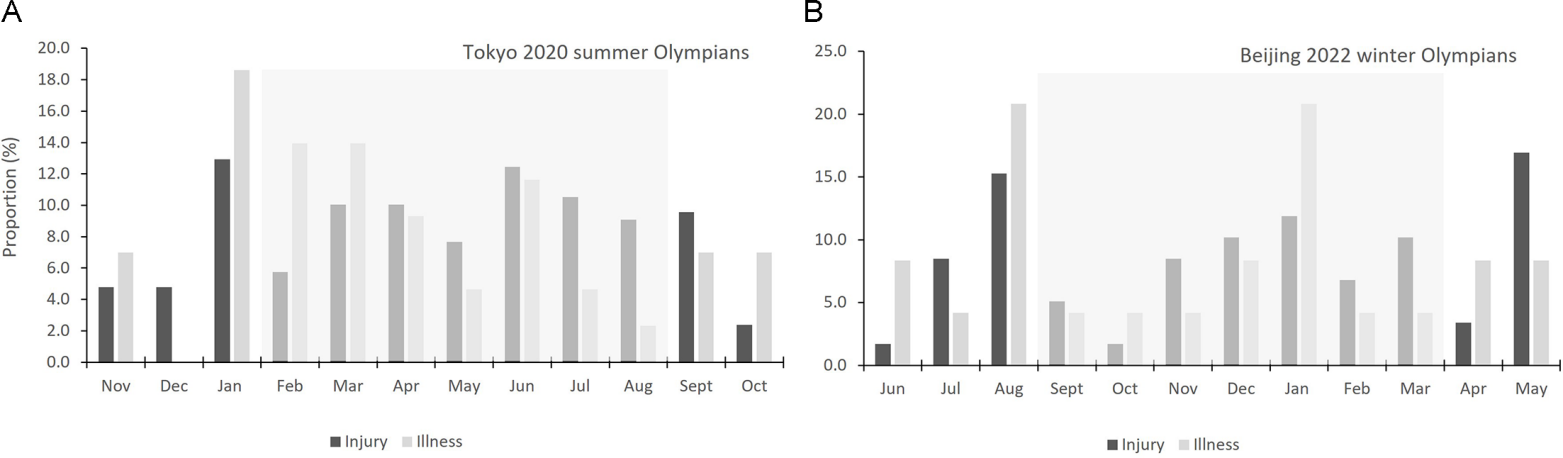
(A, B) Seasonal distribution of injury and illness for Summer and Winter Olympians, by month.

Injury and illness distribution across a season, combining the 4 years of data, are presented in figure 3A,B). The proportion of injuries and illnesses in Summer Olympians was bimodal; peaking in Jan and decreasing over the preseason (January–March) before peaking again mid-season (June) and decreasing to the end of the season. For Winter Olympians, the trend was trimodal; peaking at the end of preseason (August), mid-season (January) and again at the end of the season (May).

### Behaviours during injury and illness

**Figure 4:**
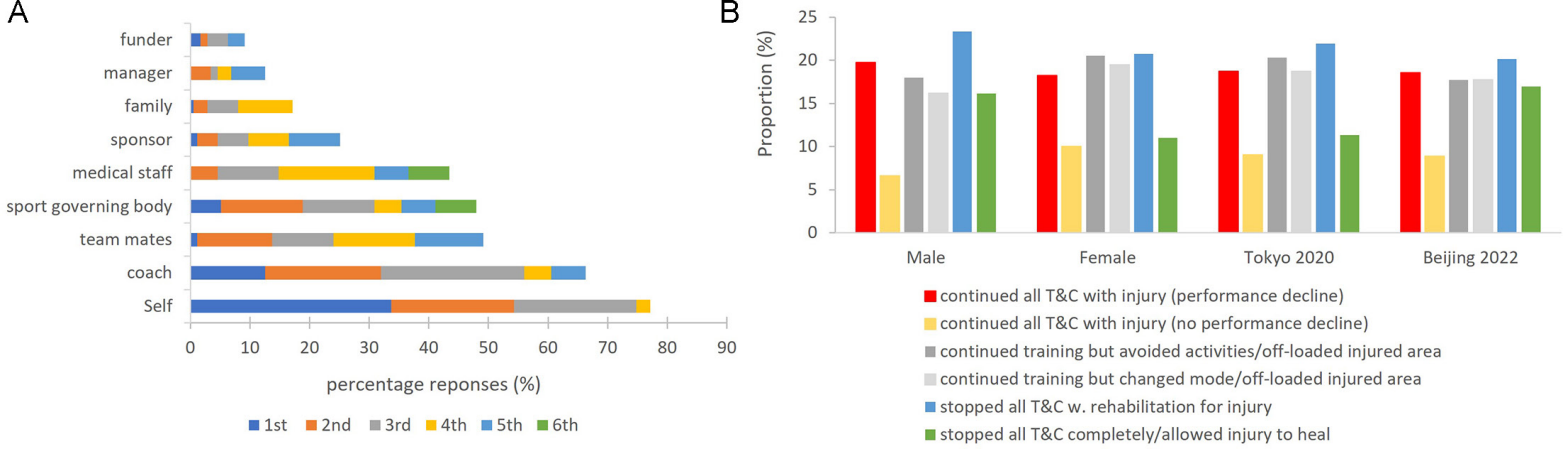
(**a**) Pressure to return from injury/illness. (**b**) Behaviours during injury/illness, by sex, Summer and Winter Olympians.

When asked what pressures they felt during injury/illness, Olympians said they were the first to put pressure on themselves to return to sport as quickly as possible. This was followed by pressure felt from coaches, teammates and their sport governing body. One-in-five Olympians continued all normal training and competition during injury/illness (with consequent performance decline) (figure 4A,B).

One-third of Olympic career-related injuries were recurrent. 43% of Olympians reported using painkillers and/or non-steroidal anti-inflammatory drugs (NSAIDs) during injury, one in five had a joint injection and one-third reported residual pain and dysfunction from previous injuries (table 3).

**Table 3:** Injury treatments and outcomes

	Male	Female	Tokyo 2020	Beijing 2022	Total
Injury recurrence	32.7% (32/98)	35.2% (77/219)	35.5% (87/245)	30.1% (22/72)	34.4% (109/317)
Pain killer use during injury	45.9% (45/98)	41.6% (91/219)	40.1% (100/245)	50.0% (36/72)	42.9% (136/317)
Joint injection during injury	20.4% (20/98)	20.5 (45/219)	22.0% (54/245)	15.3% (11/72)	20.5% (65/317)
Limited career success	45.9% (45/98)	39.3% (86/219)	40.8% (100/245)	43.1% (31/72)	41.3% (131/317)
Career ending injury	2.0% (2/98)	2.7% (6/219)	3.3% (8/245)	1.4% (1/72)	2.8% (9/317)
Lasting limitation and pain	31.6% (31/98)	32.4% (71/219)	32.2% (79/245)	30.5% (22/72)	31.9% (101/317)

Painkiller use includes reporting of analgesics and non-steroidal anti-inflammatory drugs.

## Discussion

This is the first longitudinal, global study of self-reported injuries and illnesses in Olympians during the 4-year cycle up to a Summer or Winter Olympic Games. The aims were to describe the characteristics of the cohort, the prevalence and nature of career-related injuries and illnesses, and athlete behaviours. Overall, 58% of Olympians were injured and 18% were ill; the knee, followed by shoulder and lumbar spine were the most frequently injured locations; respiratory illness was the most common illness in both Summer and Winter Olympians; Olympians felt the greatest pressure to return from injury/illness from themselves; one-quarter continued full training and competition while they were injured/ill and nearly half used painkillers; injury and illness prevalence followed similar bimodal and trimodal patterns across the season for Summer and Winter Olympians, respectively.

### Olympic career injury and illness in Tokyo 2020 and Beijing 2022 Olympians

To date, no other studies have recorded injuries or illnesses across different Summer and Winter Olympic sports in the 4 years prior to an Olympic Games. Across the preceding 4-year cycle, 59% of Summer Olympians reported a significant injury (lasting >2 weeks) and 17% a significant illness; and 56% and 24% of Beijing 2022 Winter Olympians reported a significant injury or illness, respectively. This was higher than the prevalence of injuries and illness recorded during the Tokyo 2020 Summer Olympics (9% of athletes injured, 4% ill) and the Beijing 2022 Olympics (10% injured and 4% ill), but lower than the injury rates reported in retired Olympians, where 63% reported a significant injury (lasting >4 weeks).^6^,^8^ The time periods for data collection and definitions for what becomes a recordable event vary significantly across these studies. If >4-week injury definition is used in the present study, similar to retired Olympian studies, this still equates to 49.1% of Summer Olympians and 50.6% of Winter Olympians experiencing injury. Given that two-thirds of Olympians in this study were participating in their first Olympic Games, these data show there is already a clear and significant burden of injury and illness experienced during the 4 years of preparation before their participation at the Olympics.

Although data numbers in some categories are low, early indications show the most injurious sports in the present study, such as judo, modern pentathlon, water polo, gymnastics and bobsleigh and freestyle skiing are similar to those reported previously during the Summer and Winter Olympic Games, and in retired Olympians.^6^,^8^ In addition, the most frequent location of injury was the knee, followed by shoulder and lumbar spine across Summer and Winter Olympians, which is broadly consistent with both Olympic Games and retired Olympian studies.^6^,^818^ Overall, female Olympians reported similar rates of injury compared with male Olympians. These data are similar to Olympic Games studies where occasional differences are identified in specific sports, but overall injury rates are similar. In contrast, findings from youth Olympics and retired Olympian studies do report higher injury rates for females compared with their male counterparts.^6 8 20 22^ Although results were not significant in the present study, female Winter Olympians reported higher rates of illness compared with male Winter Olympians. This is similar to the pattern observed across all previous Winter Olympics surveillance studies, since Vancouver 2010. In these studies, respiratory illness was also most common.^7 19 21 23 24^ It is well documented that the risk factors for some injuries differ for female athletes, for example ACL.^25 26^ However, there are limited data exploring the reasons for the elevated risk of illness, including respiratory illness, in female athletes. The success of COVID-19 mitigation strategies in reducing respiratory and gastrointestinal illness rates during recent major games presents a clear opportunity to address what has for some years been ‘low-hanging fruit’ in terms of illness prevention for female athletes.^6 7^ Although the present study does not include specific COVID-19 data, it is acknowledged that the pandemic likely contributed to the reported illness rates during this study’s data collection period. Conversely, COVID-19 mitigation strategies may have had a positive impact by reducing the incidence of other illnesses such as other respiratory and gastrointestinal illnesses.^6 7^

### Seasonal injury and illness patterns

Injury and illness patterns were observed to be bimodal for Summer Olympians and trimodal for Winter Olympians, indicating specific periods of heightened risk across an athlete’s season. Increases in injury risk appear to be mirrored by increases in illness risk, and vice versa. While high training and competition loads have been cited as precursors to both sports injury and illness,^27^ to date, there is no evidence of an association between illness and injury occurrence and this is the first time that the phenomenon has been seen across an athlete’s competitive season. There is evidence to suggest some injuries precede others, for example, musculoskeletal injury risk is higher during recovery from sports-related concussion due to its impact on sensorimotor function.^28^ Systemic illness has been cited to have a deleterious effect on physical function and impair aspects of athletic performance, hence this illness-related impairment on physical performance may be one explanation for a concomitant increase in injury risk.^29^ Conversely, sports stressors, including high training loads and excessive fatigue (acute and cumulative), which are risk factors for injury,^27^ are also known to depress immunity and increase infection risk.^30^ Whatever the interplay between these morbidities, the present data provide a clear opportunity for interventions at key parts of an athlete’s season—whether through modifying athlete exposures to training load, increasing rest periods, increasing illness mitigation strategies and/or identification and removal of other stressors, for example, life, work/study and travel.

### Behaviours during injury and illness

One-quarter of Olympians continued to train and compete despite being injured or ill, and nearly half used painkillers. Athletes being rest averse during injury and illness is not a new concept.^8^ Athletes who perceive greater pressure to return to sport during injury/illness, from themselves and others, are more likely to continue competing when they are hurt and to take painkillers.^831^,^33^ The rates of analgesic and NSAID use within the present study are consistent with those reported in previous elite athlete studies.^834^,^36^ Such medication use, along with other athlete behaviours and experiences (ie, higher rates of depression during injury/illness), may contribute to delayed recovery and increase the risks of recurrent health issues.^8 33 37 38^ It is important to consider how these behaviours might be addressed positively, with both the athletes themselves and their support team. A biopsychosocial approach to prevention initiatives is recommended, with greater dedicated support for athletes during periods of injury/illness-related absence.

### Strengths and limitations

This is the first worldwide study recording injuries and illnesses across a range of Summer and Winter Olympic sports during a 4-year Olympic cycle, and begins to fill the evidence gaps in our understanding of Olympian health.^6^,^8^ Surveys were distributed in eight languages and included all participating NOCs. The longitudinal nature of the survey, with follow-up every 2 years for 15 years, may have impacted the number of participants willing to get involved in the study. Hence, data in some categories may be low and impacted by sparse data bias. It is not known how many Olympians the survey emails reached, and so it is not possible to calculate an accurate response rate. Inferences at this first timepoint should be made with caution. With continuous longitudinal follow-up and as data are gathered at repeat time points, this will help strengthen findings on the existing cohort and address some of these methodological challenges. Additionally, ongoing recruitment of new participants to the cohort at future games will only improve our understanding of emerging trends and strengthen inferences around risk factors for negative health outcomes.

It is recognised there are retrospective components to some injury and illness-related questions, and hence a significant 2-week significant injury/illness definition was used to negate some of the associated recall bias.^15^ This does mean that less severe injuries/illness (ie, of less than 2 weeks duration) are not captured. When making comparisons to other studies, it is important to consider the time period for data reporting and the definition for what becomes a recordable injury or illness event. There will be a self-selection bias in the present sample, whereby Olympians who had a greater history of illness or injury may have been more likely to participate in the study. However, longitudinal data monitoring of these individuals across their entire careers may negate some of this initial bias. More detailed information on other health and well-being measures, including indices of mental health in this cohort, needs to be elucidated to build on these early findings.^39^,^41^

## Conclusions

Athletes, including first-time Olympians, already present with a significant burden of injury and illness across the 4 years before their participation at an Olympic Games. A biopsychosocial approach, helping to provide additional support to athletes and addressing behaviours during injury and illness is needed. There appears to be a concomitant relationship between injury and illness patterns across a season, suggesting the need for a coordinated approach at key time points in the athlete’s year. Lessons learnt from major sporting events in preventing respiratory illnesses such as COVID-19 would offer quick and easy solutions to reducing these health burdens at different points in the athletes’ season.

## Supplementary material

10.1136/bmjsem-2025-002545 online supplemental figure 1

## Data Availability

All data relevant to the study are included in the article or uploaded as supplementary information.
