# Supplementary figures and images for: Insights from the first IOC Olympian Health Cohort: injury and illness in Olympians preparing for the Tokyo 2020 Summer and Beijing 2022 Winter Olympic Games
Source: BMJ Open Sport Exerc Med. 2025 Sep 21;11(3):e002545. doi: 10.1136/bmjsem-2025-002545 (PMC12458888; doi:10.1136/bmjsem-2025-002545)

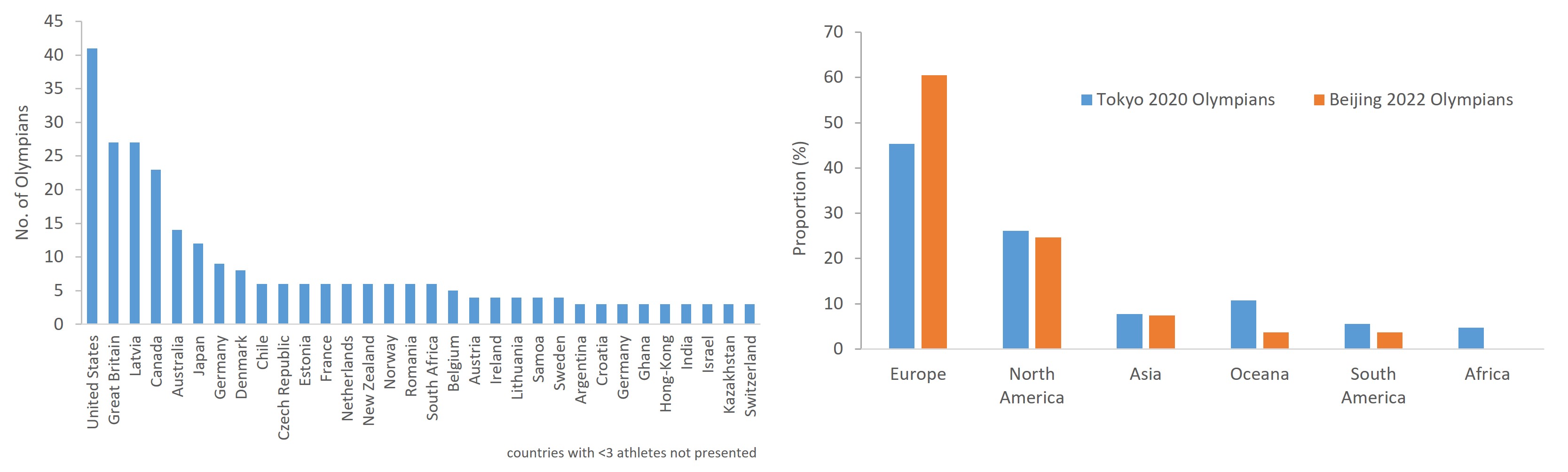

Supplement: online supplemental figure 1 [file bmjsem-11-3-s001.jpg]
